# New Pressure-Sensitive Acrylic Adhesives for Low Energy Substrates Prepared via UV-Induced Telomerization with a Fluorine-Based Telogen

**DOI:** 10.3390/ma15238667

**Published:** 2022-12-05

**Authors:** Agnieszka Kowalczyk, Agata Kraśkiewicz, Krzysztof Kowalczyk

**Affiliations:** Faculty of Chemical Technology and Engineering, West Pomeranian University of Technology, Piastów Ave. 42, 71-065 Szczecin, Poland

**Keywords:** pressure-sensitive adhesives, telomerization, UV-crosslinking, adhesion, polyethylene

## Abstract

Novel pressure-sensitive adhesives (PSA) for low energy substrates were prepared by a solvent-free UV-initiated telomerization process using n-butyl acrylate, butyl methacrylate, and lauryl methacrylate (LMA), with trifluoroethanol (TFEtOH) as a telogen, and acylphosphine oxide (APO) as a radical photoinitiator. A crosslinking monomer (an aliphatic urethane acrylate, L9033) and a radical UV-photoinitiator (α-hydroxyalkylphenone) were also tested as components of the adhesive compositions. The influence of LMA and TFEtOH on the UV-phototelomerization process kinetics and the physicochemical features of the obtained fluorotelomers, as well as the concentration of L9033 on the PSA adhesion to a polyethylene surface, were investigated. FT-IR results indicated that the fluorine groups were successfully introduced into the telomer structure. The highest adhesion relative to a polyethylene substrate (12.3 N/25 mm), and the highest hydrophobicity (with a contact angle of 95° for a water/PSA system) were observed for adhesives based on a telomer syrup containing 5 wt. parts of TFEtOH and 30 wt. parts of LMA (per 100 wt. parts of the monomer mixture). Additionally, it was revealed that a higher aliphatic urethane acrylate content and a higher UV dose increased the adhesion feature.

## 1. Introduction

Pressure-sensitive adhesive (PSA) tapes are broadly used in the construction, medical, automotive, and aerospace industries, as well as in many others [1,2]. In the construction of equipment, they are invaluable for holding and positioning conductors, preventing relative movement, and identifying parts [3].

Achieving the desired adhesion of PSA to low surface energy substrates, such as polyethylene (PE), isotactic polypropylene, or polytetrafluoroethylene, usually requires surface pretreatment, i.e., corona discharge, plasma, or flame treatment of the bonded surface just prior to the application of the adhesives [4,5]. Alternatively, a compatible polymer solution (primer) can be applied onto the substrate [6,7]. In the last decade, several patents for the preparation of pressure-sensitive adhesives for low energy surfaces have been issued. The innovative solutions mainly consist of the use of (i) plasticizers (e.g., polyglycol ethers, phosphate esters) at the preparation stage of the polyacrylate adhesive composition [8], (ii) components with long aliphatic chains in combination with (meth)acrylate derivatives of polyethylene glycol (e.g., butenoxydiethylene glycol methacrylate) [9], or (iii) special comonomers, such as isobornyl acrylate, isooctyl acrylate [10] or stearyl acrylate [11]. Another approach is to use fluorine-containing monomers (F-monomers), such as 2,2,2-trifluoroethyl methacrylate [12], 2,2,3,4,4,4-hexafluorobutyl acrylate [13], hexafluorobutyl methacrylate, and dodecafluoroheptyl methacrylate [14]. It is known that fluoropolymers possess low surface energy, as well as outstanding chemical resistance, weather stability, a low coefficient of friction, and a low dielectric constant. These properties come from the special electronic structure of the fluorine atom, the stable carbon–fluorine covalent bonding, and the unique intramolecular and intermolecular interactions between the fluorinated polymer segments and the main chains [15,16]. Most fluoropolymers are produced by classical radical polymerization in aqueous systems [17]; however, one of the most interesting production strategies is telomerization. This method of synthesis allows for the prediction of the degree of polymerization, the structure of the product, and the mechanism of the reaction. In contrast to polymerization, telomerization, introduced for the first time by Hanford in 1942 [18], usually leads to low molecular weight polymers (telomers), or even to monoadducts, with well-defined end-groups. The products are obtained by a reaction between a telogen or a transfer agent (X–Y), and one or more molecules of a polymerizable compound M (taxogen/monomer) exhibiting ethylenic unsaturation under radical polymerization conditions. Telomerization can be initiated via various processes: photochemically (in the presence of UV-light), in the presence of a radical initiator or redox catalysts, thermally, or initiated by X- or γ-rays. The first investigation of photochemically induced telomerization (an addition of alcohol to hexafluoropropene) was described by Haszeldine et al. Photochemically induced processes for the preparation of fluorotelomers rely on the reaction of fluoroolefins (e.g., VDF) and various telogens (e.g., iodoalkanes or brominated methane-derivated telogens: CFBr_3_). The telomerization of VDF has been extensively investigated by many authors. However, C-F type telogens are not used in this type of process due to the high bond dissociation energy of the C-F bond (486 kJ/mol) [19].

This paper describes an innovative method for the preparation of novel pressure-sensitive acrylic adhesives (PSA). Fluorooligomers (fluorotelomers)—used as adhesive binders—were prepared via the UV-photoinduced telomerization of n-butyl acrylate, butyl methacrylate, and lauryl methacrylate in the presence of trifluoroethanol (as a telogen) and acylphosphine oxide (as a radical photoinitiator). Besides the fluorotelomer syrup, the PSA contained a crosslinking monomer (an aliphatic urethane acrylate) and an additional radical photoinitiator (α-hydroxyalkylphenone). Specific features of the syrups and the UV-photocurable adhesive compositions, as well as the adhesion of the UV-photocured PSA to a polyethylene surface, were analyzed. It was revealed that UV-phototelomerization can be an environmentally acceptable way (rapid, organic solvent-free, low energy cost) to produce new fluorooligomers for solvent-free PSAs with excellent adhesion to low energy substrates.

## 2. Materials and Methods

### 2.1. Materials

The following components were used for the preparation of the fluorotelomer syrups:

- Taxogens/monomers: n-butyl acrylate (BA), butyl methacrylate (BMA), lauryl methacrylate (LMA) (BASF, Ludwigshafen, Germany);
- Telogen: trifluoroethanol (TFEtOH, POCh, Gliwice, Poland);
- Radical UV-photoinitiator: bis(2,4,6-trimethylbenzoyl)-phenylphosphineoxide (APO; Omnirad 819, IGM Resins, Waalwijk, The Netherlands).

The PSA compositions were prepared using the fluorotelomers syrups and:

- Crosslinking monomer: aliphatic urethane acrylate (L9033; Laromer 9033, Ludwigshafen, Germany);
- Radical UV-photoinitiator: hydroxycyclohexylphenyl ketone (HAP; Omnirad 184, IGM Resins, Waalwijk, The Netherlands).

**Figure 1:**
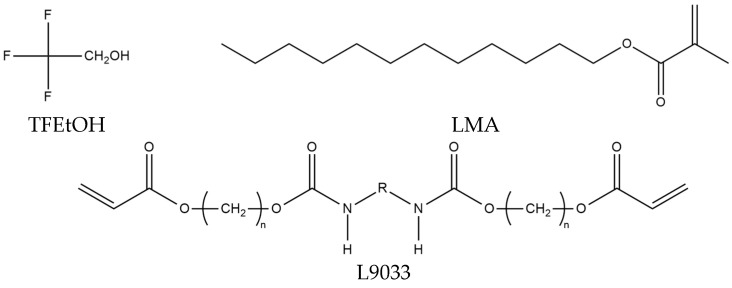
Chemical structures of selected components.

The chemical structures of the selected components are shown in Figure 1.

### 2.2. Methods

#### 2.2.1. Fluorotelomers Syrups Preparation

**Figure 2:**
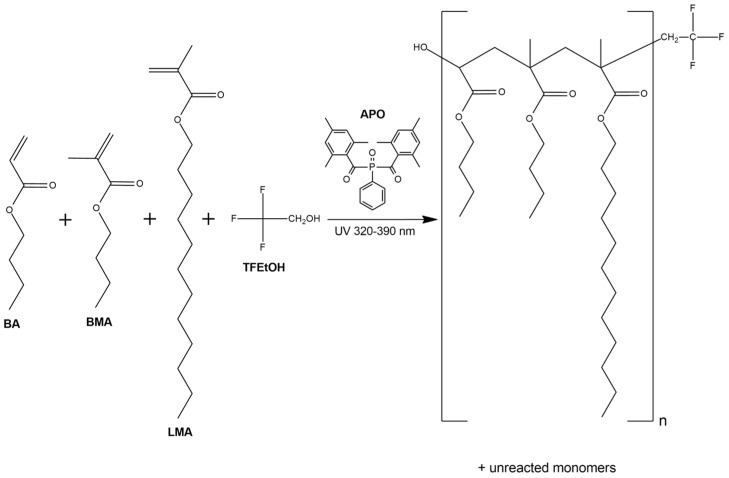
The schematic reaction of the UV-induced telomerization process.

The telomerization (cotelomerization) process was carried out in the presence of an inert gas (Ar) at 25 °C for 15 min in a glass reactor (250 mL) equipped with a mechanical stirrer, thermocouple, and a cooler. The UV-LED strip (λ = 390 ± 5 nm; MEiSSA, Warsaw, Poland) was used as a UV light source. The UV-irradiation inside the reactor (15 mW/cm^2^) was controlled using the SL2W UV-radiometer (UV-Design, Brachttal, Germany). The schematic reaction of the UV-induced telomerization process is shown in Figure 2.

#### 2.2.2. Pressure-Sensitive Adhesives Preparation

The prepared fluorotelomers syrups (FT) or acrylic copolymers syrups (AS; reference systems without fluorotelogen produced by a bulk photopolymerization process) were mixed with the aliphatic urethane acrylate (2, 4, or 6 wt. parts/100 wt. parts of the syrup) and the HAP photoinitiator (1 wt. part/100 wt. parts of the syrup). The adhesive compositions were stored for 24 h in a dark place; then, they were applied onto a polyester foil and UV-irradiated (the UV doses of 4, 6, 8, 9, or 10 J/cm^2^) using a UV-ABC medium-pressure mercury lamp (Hönle UV-Technology, Gräftingen, Germany). The UV exposition was controlled by the Dynachem 500 radiometer (Dynachem, Westville, IL, USA). Grammage of the UV-crosslinked PSA layers was 60 g/m^2^. The composition of the AS and FT systems is shown in Table 1.

#### 2.2.3. Characterization of the Fluorotelomers Syrups

The dynamic viscosity of the FT and AS syrups was measured at 25 °C using the DV-II Pro Extra viscometer (spindle #6 or #7, 50 rpm; Brookfield, New York, NY, USA). The solids content was determined using a thermobalance (Radwag, Radom, Poland); samples (ca. 2 g) were heated in an aluminum pan at 105 °C for 4 h. Gel permeation chromatography (GPC) was used for determination of the molecular masses (Mw, Mn) and the polydispersity of the fluorotelomers (samples were heated at 140 °C for 4 h before the test in order to remove unreacted monomers). Moreover, the kinetics studies of the UV-induced telomerization process were realized at 25 °C by the photo-DSC method (the differential scanning calorimeter with UV equipment; Q100, TA Instruments, New Castle, DE, USA). During the measurements, samples (5 mg) were UV-irradiated (320–390 nm) with the intensity of 15 mW/cm^2^ in an Ar atmosphere. The polymerization rate (*R_p_*, %/s) and the conversion of double bonds (*p*, %) were calculated according to Equations (1) and (2), respectively:

(1) Rp=(dHdt)H0 [%s]

(2) $$p=\frac{∆H_{t}}{∆H_{0}}*100\;\left[\%\right]$$

where: *dH*/*dt*—recorded heat flow during UV-irradiation, *H*_0_—the theoretical heat value for the complete degree of conversion (∆*H* = 78.0 kJ/mol for acrylates and ∆*H* = 54.8 kJ/mol for methacrylates), and ∆*H_t_*—reaction heat evolved at t-time [20].

The presence of fluorine atoms in the telomeric syrups and in the dry telomerization products was monitored using a Fourier transform infrared spectroscope with ATR accessories (Nicolet 380, Thermo Scientific, Waltham, MA, USA). An analysis of the FTIR spectra consisted of determining the presence of an absorption band for the polyphased C-F moieties (1400–1100 cm^−1^) [12]. The reference sample was the TFEtOH telogen.

#### 2.2.4. Characterization of UV-Crosslinked PSA

The adhesion of UV-crosslinked PSA to a polyethylene substrate (PE) was determined at the angle of 180°, according to the AFERA 5001 standard developed by the European Association for Testing Adhesives for the Automotive Industry (AFERA) using the Z010 testing machine (Zwick/Roell, Ulm, Germany). PSA samples (175 mm × 25 mm) were applied onto a degreased PE plate and pressed with a rubber roller (2 kg) in order to improve the wetting of the substrate by the tested adhesive. The adhesion test was performed 20 min after the film application, with a peeling speed of 300 mm/min. Five measurements for each PSA were performed.

The contact angles for water/PSA systems were measured with the EO 300A goniometer (Surface & Electro-Optics, Suwon-si, Republic of Korea). A single drop of distilled water was placed on the PSA surface. The contact angle was observed after 5 s on the PSA surface, using a high-speed camera (1000 frames/s).

The glass transition temperature (T_g_) of the PSA was determined using differential scanning calorimetry (DSC Q100; TA Instruments, New Castle, DE, USA). Samples (ca. 10 mg) of the UV-crosslinked PSA were placed in hermetic aluminum pans and heated from −90 °C to 100 °C at the heating rate of 10 °C/min. The T_g_ values were determined as a temperature value of the endothermic inflection point. 

## 3. Results and Discussion

### 3.1. Kinetics of the UV-Phototelomerization Process

**Figure 3:**
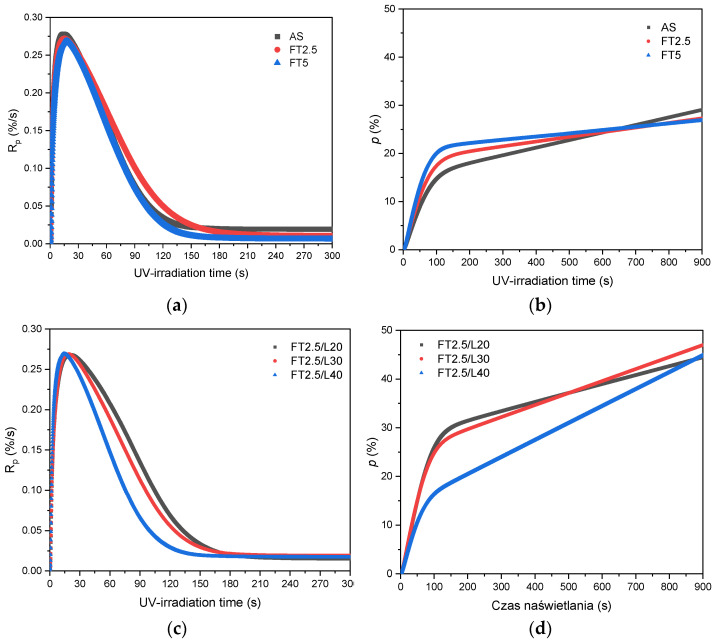

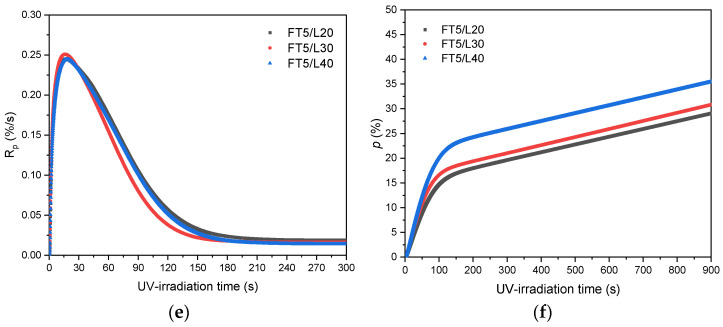
Reaction rate (*R_p_*) and conversion of double bonds (*p*) for the UV-phototelomerization process of BA, BMA, and LMA in the presence of different fluorotelogen doses (**a**,**b**), with 2.5 wt. parts of the fluorotelogen and different LMA content (**c**,**d**), or with 5 wt. parts of the fluorotelogen and different LMA content (**e**,**f**) (I_0_ = 15 mW/cm^2^; 320–390 nm; Ar).

At the beginning, the influence of the fluorotelogen (TFEtOH) and lauryl methacrylate (LMA) concentrations on the UV-photochemically induced telomerization process of the selected monomer (taxogen) systems was investigated using the photo-DSC method. The research was performed for the systems containing BA (55 wt. parts), BMA (15 wt. parts), LMA (30 wt. parts), and the APO photoinitiator (0.5 wt. part/100 wt. parts of the monomer mixture) (Figure 3a,b). Content of TFEtOH was varied. In the remaining figures, data for the systems showed different amounts of LMA, and 2.5 (Figure 3c,d) or 5 wt. parts of TFEtOH/100 wt. parts of the monomer mixtures (Figure 3e,f) are presented. 

It was found that the reaction (i.e., the UV-photopolymerization process) in the telogen-free systems (AS) proceeded slightly faster than in the others (*R_p_* = 0.28%/s), and after ca. 130 s of exposure, it decreases to a low speed (0.03%/s). Generally, the more telogen content, the lower the maximum reaction speed value, and the maximum reaction speed was recorded slightly later a delay in the reaction due to an additional step of C-H bond cleavage in the fluorinated alcohol). However, it is visible (Figure 3a) that the more telogen in the system, the faster the reaction ends (the reaction rate for FT5 is practically zero after 130 s of exposure). Most likely, this is caused by a termination process resulting from the transfer of the kinetic chain to the telogen molecules. It is known that alcohols are involved in the chain transfer by hydrogen abstraction from the α-carbon, or (rarely) by hydrogen abstraction from the hydroxyl group. Thus, the conversion of unsaturated bonds (after 15 min of UV-irradiation) is the highest in the AS-type systems (29%), and slightly lower in these with TFEtOH (approx. 27%). Figure 3c,d shows the effect of lauryl methacrylate on the UV-phototelomerization rate and conversion of unsaturated bonds in the compositions containing 2.5 wt. parts of TFEtOH. The maximum *R_p_* value and the time required to reach this value are similar for all samples (regardless of the LMA content). However, the more LMA in the system, the slower the reaction rate, which is consistent with the results in the literature. At 40 wt. parts of LMA, the reaction was practically complete after 120 s of UV-irradiation (160 s for 20 wt. parts of LMA). However, the conversion values of the monomers after 15 min of the reaction were similar (40–45%). Figure 3e,f shows the effect of the LMA concentration on the UV-phototelomerization rate and conversion of unsaturated bonds in the systems containing 5 wt. parts of TFEtOH. In comparison to the FT2.5 systems, the highest (or the lowest) conversion was noted at 40 (or 20) wt. parts of LMA. The reaction was moderate at 30 wt. parts of LMA. Summarizing this part of the research, it should be noted that LMA has a more significant impact on the UV-phototelomerization process than does TFEtOH. Generally, the more fluorotelogen molecules in the system, the slightly lower the monomer conversion.

### 3.2. The Physicochemical Properties of the Fluorotelomers Syrups

**Figure 4:**
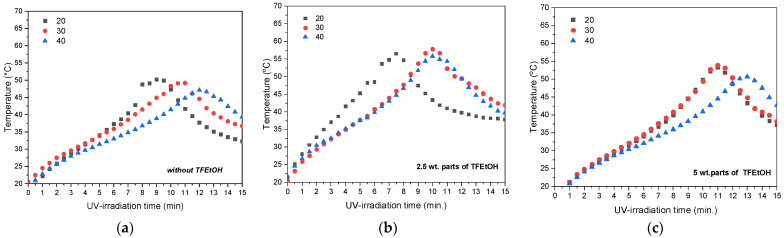
Temperature during the UV-phototelomerization process (20, 30, or 40 wt. parts of LMA and 0 (**a**), 2.5 (**b**), or 5 wt. parts of the fluorotelogen (**c**).

The course of the UV-phototelomerization process in the glass reactor (at desired mixing speed of the reactants) was investigated by the registration of the mixture temperature; the thermographs for the systems with different content of lauryl methacrylate (20, 30 or 40 wt. parts) and the variable content of TFEtOH (AS; FT2.5 and FT5) are presented in Figure 4.

The presented thermographs confirm that the higher lauryl methacrylate concentration generally slows down the UV-phototelomerization process because the recorded temperature peak occurred much later (after 12 min of exposure for the system with 40 wt. parts of LMA and without fluorotelogen), while the temperature value was relatively low (<50 °C; Figure 4a). This tendency was also maintained in the systems with fluorotelogen; however; the compositions with 2.5 wt. parts of TFEtOH reached a slightly higher maximum temperature value (55–60 °C). Corresponding to the photo-DSC results; the reaction rates for these samples (Figure 3c,d) were higher than for the systems with 5 wt. parts of the fluorotelogen. Nevertheless, it should be noted that the reactions in the glass reactor were carried out with the different photoinitiator contents (0.45–0.65 wt. part; Table 1). Research (data not presented) indicates that a higher lauryl methacrylate content in the reaction mixture requires a greater amount of the photoinitiator in order to observe an increment in the telomer syrup viscosity. The later component concentration in the systems was experimentally selected to obtain telomeric solutions with similar fluorotelomers content (i.e., with similar SC values) and with a viscosity value >5 Pa·s, conditions which are suitable for the modification of and application onto the PSA polyester carrier. Selected physicochemical properties of the prepared telomer syrups (i.e., the dynamic viscosity and molecular weights of the telomers) are presented in Table 2

**Figure 5:**
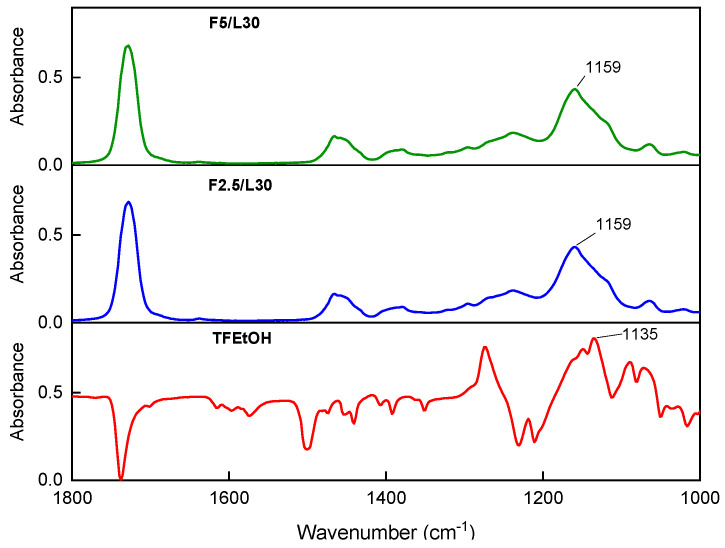
FTIR spectra of the fluorotelogen and fluorotelomers.

As can be observed, the SC values for all systems were similar (approx. 70%). However, this parameter represents the conversion of the reactants; the recorded values are higher than those for the conversion of the unsaturated bonds (*p*) calculated using the photo-DSC data. We have previously reported [21] that the noted discrepancy resulted from the fact that the components were mechanically mixed during the UV-phototelomerization process in the reactor (no mixing during the photo-DSC measurements). Moreover, in some systems the photoinitiator concentration was higher than in the samples investigated by photo-DSC. Interestingly, in the case of telogen absence and at a low concentration of the APO photoinitiator and lauryl methacrylate (only 20 wt. parts), it is not possible to prepare a telomer syrup (sample AS/L20); the system gelled during the UV-phototelomerization process. It was revealed that a higher lauryl methacrylate content in the reaction mixture causes a reduction in the molecular weights and polydispersity (ca. 5) of the copolymers and fluorotelomers. Nevertheless, the incorporation of 2.5 wt. parts of fluorotelogen more significantly lowers the molecular weights of the products (Mw from ca. 100,000 to ca. 20,000 g/mol); further decrements of Mw and Mn were also noted at the higher fluorotelogen concentration (FT5). The fluorotelogen addition markedly lowers the polydispersity value of the telomers (up to 1.9–1.7), which is characteristic for a controlled polymerization processes [22]. The presence of CF_3_ groups in the obtained telomerization products (from the TFEtOH) was confirmed by the FTIR technique (Figure 5); an absorption band at the wavenumber of 1135 cm^−1^ (corresponding to the C-F stretching vibration [12]) was visible in the spectra of the fluorotelogens. For both telomers (i.e., the solids of the FT2.5 and FT5-type samples), this characteristic peak was also observed, but was slightly shifted by ca. 20 cm^−1^. The band shift is attributed to the presence of hydroxyl groups (at the ends of the telomers) [23].

### 3.3. Properties of UV-Crosslinked PSA Based on Fluorotelomers

**Figure 6:**
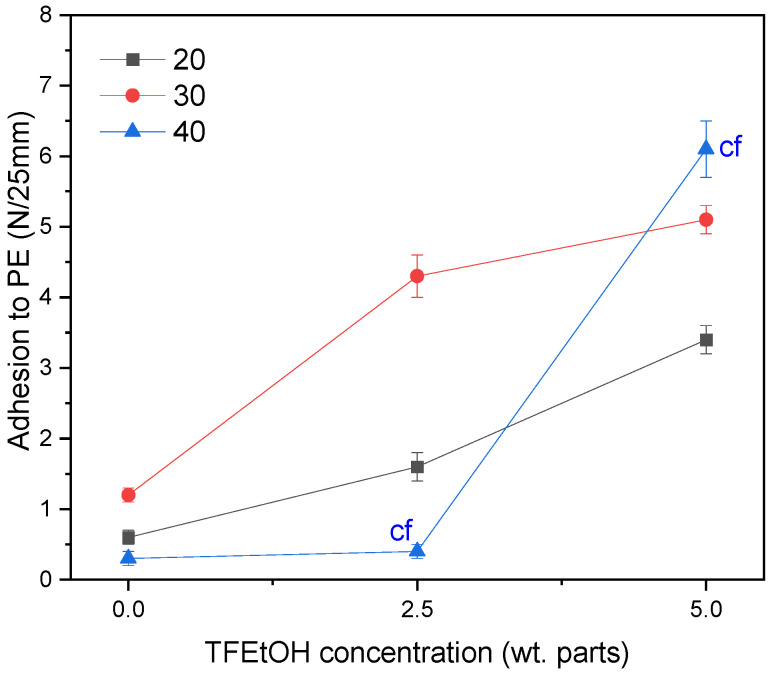
Adhesion to PE of PSA (with different fluorotelogen and LMA content, 2 wt. parts of aliphatic urethane acrylate/100 wt. parts of a syrup; UV dose of 8 J/cm^2^) (cf—cohesive failure).

As described above, the concentration of the fluorotelogen, lauryl methacrylate, and the photoinitiator strongly affects the molecular weights of the fluorotelomers as well as the monomers conversion (i.e., the amount of unreacted monomers remaining in the syrups). These parameters—mainly the content of the unreacted monomers, which then react with the multifunctional monomer and the UV-photoinitiator (HAP) during the UV-crosslinking process of PSA—also influence the adhesive properties of the tapes. It should be noted that the adhesive compositions (based on the fluorotelomers or acrylic copolymers, as the reference systems) used for PSA preparation were characterized by a similar monomers conversion (SC approx. 70 wt.%); however, the molecular weights of the fluorotelomers (and the acrylic copolymers) were varied. Nevertheless, the main difference between the tested PSA compositions was the content of (terminal) fluorine atoms. The adhesion values of the UV-irradiated PSA samples (2 wt. parts of L9033/100 wt. parts of syrups; UV dose of 8 J/cm^2^) to a polyethylene substrate are presented in Figure 6.

**Figure 7:**
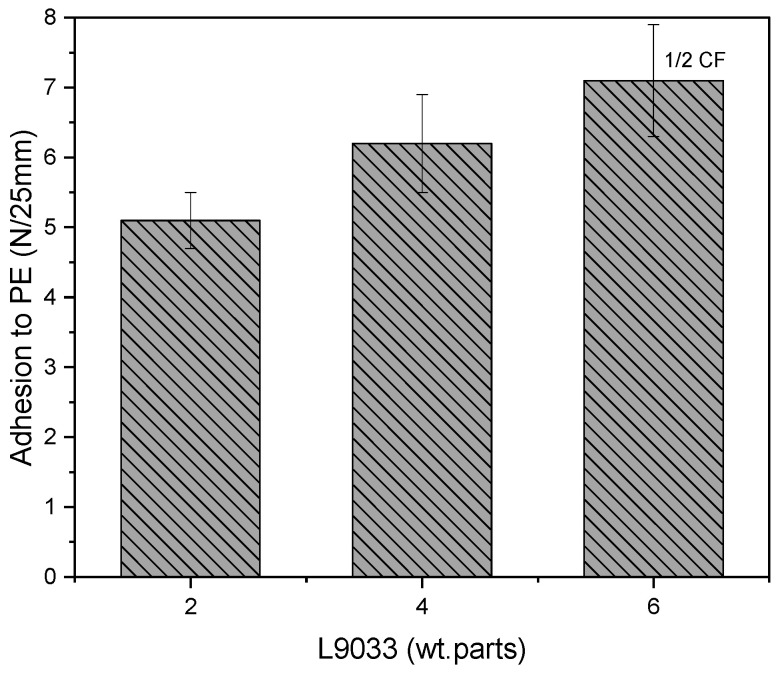
Adhesion to PE of PSA (with the FT5/L30 syrup; UV dose of 8 J/cm^2^) in relation to aliphatic urethane acrylate content; ½ CF—partial cohesive failure.

As can be seen, the values of adhesion to the PE surface for the fluorine-free samples (i.e., PSA, based on the AS syrups) are relatively low (<1.5 N/25 mm). In the case of PSA with the FT syrups, that parameter value was markedly increased (up to 5 N/25 mm for PSA, based on FT5/L30 and 6 N/25 mm for PSA with FT5/L40). This shows that the fluorotelomers—similarly to the fluoropolymers—exhibit low surface energy. This feature results from a special electronic structure of the fluorine atom, the stable carbon-fluorine covalent bonding, and the unique intramolecular and intermolecular interactions between the fluorinated polymer segments and the main chains [24]. Nevertheless, it can be seen that adhesion to PE significantly depends on the LMA concentration in the syrups; PSA based on the fluorotelomers with 30 wt. parts of LMA exhibited higher adhesion than the sample with 20 wt. parts of LMA; however, the results for the system with 40 wt. parts of LMA were strongly affected by the TFEtOH concentration (the above described phenomenon was observed only for the FT5-based samples). Additionally, it should be noted that the PSA with FT2.5/L40 or FT5/L40 exhibited cohesive failure during the adhesion test. This was caused by the relatively lowest Mw and Mn values of these fluorotelomers (Table 2). To demonstrate the influence of the telogen and LMA concentration on the adhesion of PSA to the PE surface, the investigated systems contained the smallest amount of the crosslinking monomer (only 2 wt. parts of L9033/100 wt. parts of syrups). It is known that this component—due to its chemical structure (a long aliphatic chain)—is used in adhesive and coating compositions intended for surfaces with low surface energy. Figure 7 shows the effect of the L9033 concentration on the PSA adhesion to PE (the same UV dose was applied at the crosslinking stage, i.e., 8 J/cm^2^).

**Figure 8:**
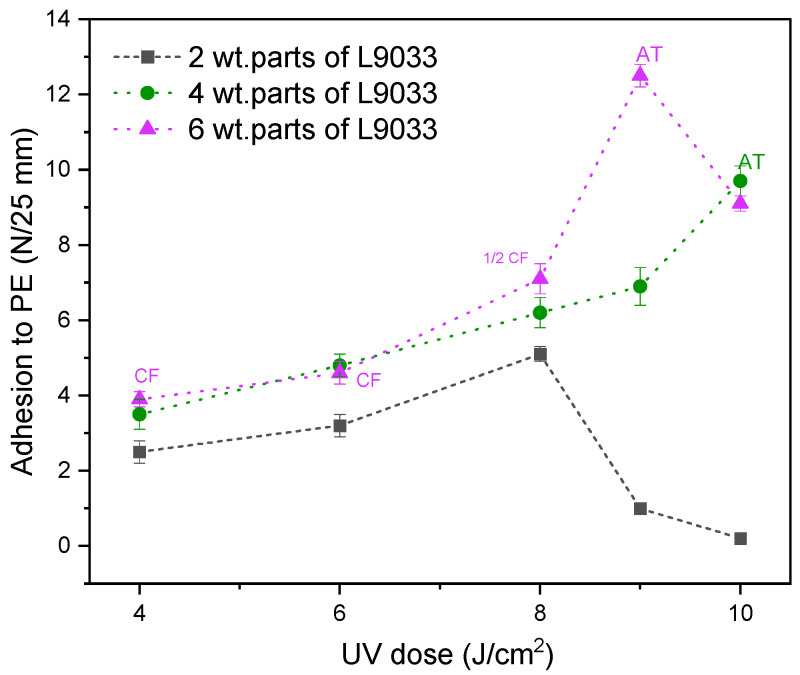
Adhesion of PSA with FT5/L30 to PE in relation to UV dose and aliphatic urethane acrylate content (CF—cohesive failure; ½ CF—partial cohesive failure; AT—adhesive transfer to the PE surface).

Generally, the increasing content of the L9033 crosslinking monomer improves the adhesion of PSA (up to ca. 7 N/25 mm for PSA with 6 wt. parts of L9033). Unfortunately, in the case of the highest L9033 concentration, partial cohesive failure of the adhesive film was noted (caused by the low crosslinking density of the PSA systems due to the insufficient UV dose). Thus, the influence of the UV-irradiation dose (during the UV-photocrosslinking stage) on the adhesion of PSA with the FT5/L30 syrup and various crosslinking monomer contents was investigated (Figure 8).

**Figure 9:**
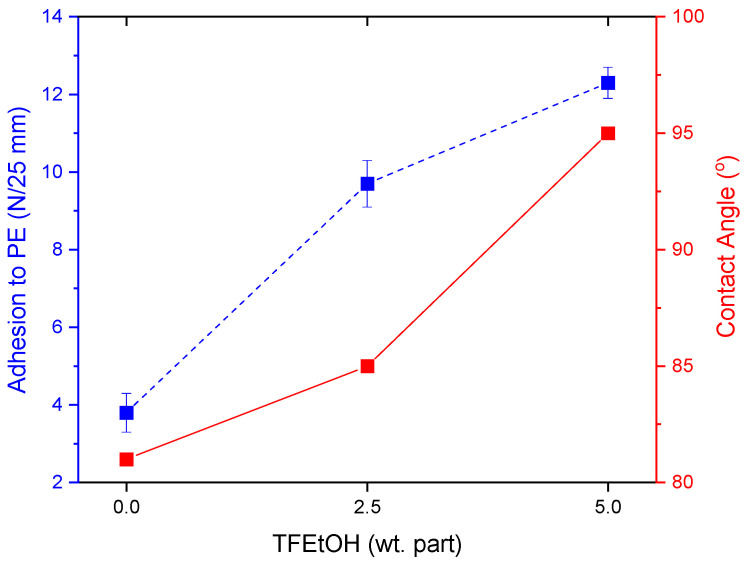
Adhesion of PSA to PE and the contact angle for a PSA/water system in relation to the fluorotelogen concentration in the PSA sample (PSA with 6 wt. parts of L9033 and syrups containing 30 wt. parts of LMA).

**Figure 10:**
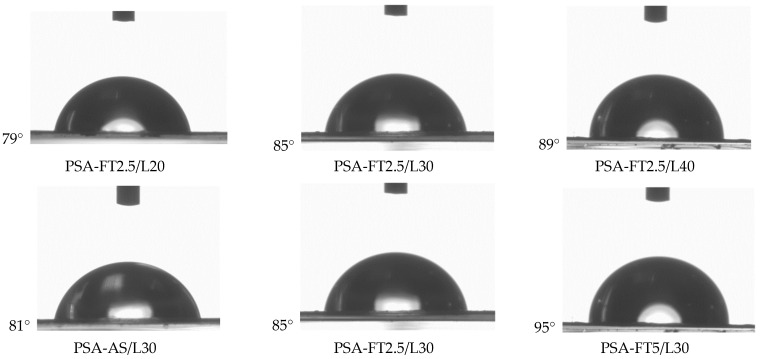
Contact angle values for water on the PSA surfaces in relation to LMA and fluorotelogen content in the FT-type syrups (PSA with 6 wt. parts of L9033; UV dose of 8 J/cm^2^).

It can be claimed that the adhesion of PSA to PE increases with an increasing UV dose (but only to a specific value of the later parameter, e.g., 8 J/m^2^ for the PSA system with 2 wt. parts of L9033). Higher adhesion values were also recorded for the samples with a higher L9033 crosslinking monomer concentration. It should be noted that the mentioned PSA samples (2 wt. parts of L9033) after UV-crosslinking using 9 or 10 J/cm^2^ were characterized by a very low adhesion to PE (<2 N/25 mm), which was probably caused by (i) too low a content of the L9033 monomer (its long aliphatic chains are supposed to increase adhesion to low energy surfaces), and (ii) too high a crosslinking density of the system (an effect of a dry adhesive film). In the case of PSA with 4 wt. parts of L9033, a very good adhesion to PE was reached (10 N/25 mm at 10 J/cm^2^), and the samples were properly cross-linked (cohesive damage was not observed). On the other hand, the transfer of the adhesive film to the PE surface was noted for this system; this means that real adhesion to this substrate was greater than 10 N/25 mm (this value represents the adhesion of the PSA system to the polyester carrier). The adhesive film transfer effect was also recorded for PSA with the highest content of the L9033 monomer (6 wt. parts), UV-irradiated by 9 J/cm^2^ (i.e., 12.3 N/25 mm). This sample also reached the highest adhesion result in the series of tests presented. Thus, for the similar samples (PSA with 6 wt. parts of L9033 monomer, with different content of the fluorotelogen), the contact angle tests were realized (Figure 9). The results presented in this paper proved that an increase in fluorotelogen concentrations improves the adhesion of PSA to low energy surfaces. The study of the contact angle revealed that the telogen presence also increases the hydrophobic nature of the surface of the cross-linked PSA films. In the case of the system exhibiting the relatively highest adhesion value (12.3 N/25 mm), the wetting angle was also very high (95°). Generally, the contact angle increases with the increasing content of LMA in the system (from 79° for PSA with FT2.5/L20 to 89° for PSA with FT2.5/L40; Figure 10).

**Figure 11:**
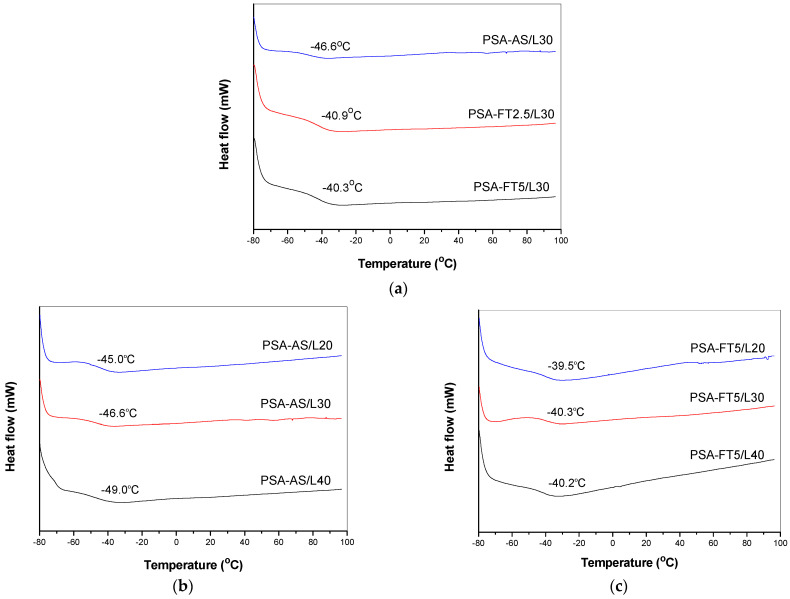
DSC thermograms for PSA (6 wt. parts of L9033; UV dose of 8 J/cm^2^) containing the syrups with 30 wt. parts of LMA and different fluorotelogen contents (**a**), without fluorotelogen and with different LMA contents (**b**), with 5 wt. parts of the fluorotelogen and different LMA contents (**c**).

The glass transition temperature (T_g_) is a critical parameter determining the usage of a polymer in a PSA system. It is known that the T_g_ for acrylic-based PSA tapes must be in the range from −40 °C to −60 °C [25]. The T_g_ is a function of molecular weight (Mn), as well as other chemical and macromolecular characteristic of the polymer, e.g., chemical composition and crosslink density [26]. Figure 11 shows the DSC thermograms with marked values of the glass transition temperature.

Interestingly, the fluorotelomer-based PSA samples (FT2.5/L30 or FT5/L30) exhibited almost identical glass transition temperature values (−40 °C); they were only slightly higher (by 7 °C) than the T_g_ of the acrylate syrups-based systems (Figure 11a). These components were characterized by different Mn values (Table 2); in the fluorotelogen-free sample, the molecular weight of the linear acrylate copolymers was much higher (22,000 g/mol) in relation to PSA with the FT-type syrups (1400 and 1300 g/mol, respectively). On the other hand, the SC values were similar (ca. 70%; Table 2). It can be concluded that the cross-linking density was higher in the fluorotelomer-based PSA materials (the photocrosslinking process of the unreacted monomers and the crosslinking monomer is easier in the presence of short-chain telomers). Finally, this causes the low mobility of the (crosslinked) polymer chains and increases the T_g_ value. The content of the methacrylate monomer (with long aliphatic chains) in PSA also influences this parameter value. The known T_g_ of poly(lauryl methacrylate) is ca. −65 °C. The presented tests results confirmed that LMA lowers the T_g_ of the PSA systems, based on the fluorotelogen-free syrups (Figure 11b). Interestingly, T_g_ for the PSA tapes with the fluorotelomers (5 wt. parts) was ca. −40 °C (regardless of the LMA content; Figure 11c). Likely, this was caused by the above-mentioned high cross-linking density of these samples (they are characterized by the low molecular weight values, which facilitates the photocrosslinking process of the adhesive films).

## 4. Conclusions

Syrups of acrylic telomers with terminal F atoms and a long aliphatic backbone (C_11_) were prepared via a UV-phototelomerization process using n-butyl acrylate, butyl methacrylate, lauryl methacrylate (LMA), and trifluoroethanol (TFEtOH, as a telogen), and then compounded with the crosslinking monomer (an aliphatic urethane acrylate, L9033) and the UV-photoinitatior (hydroxycyclohexylphenyl ketone, HAP). The obtained compositions were used for the creation of pressure-sensitive adhesives (PSA) intended for use with low energy substrates (e.g., PE). The main conclusions are as follows:

- The addition of the telogen lowers the maximum reaction rate of the photopolymerization process only slightly; however, it significantly reduces molecular weights and polydispersity of the telomers. The kinetics of the UV-phototelomerization process is also influenced by the qualitative/quantitative composition of the monomer mixture.
- A greater LMA concentration reduces the maximum temperature peak value during the phototelomerization process in a glass reactor with continuous mixing of the reactants, and slightly lowers the molecular weights of the fluorotelomers.
- The adhesion of the PSA tapes to the low energy surface (PE) can be significantly increased by a higher dose of the aliphatic urethane acrylate and a higher UV dose.
- The excellent adhesion of PSA to PE (12.3 N/25 mm), as well as a high contact angle (95° for a water/PSA system), were noted for the sample based on a telomer containing 5 wt. parts of the telogen (per 100 wt. parts of the monomers mixture), the intermediate LMA amount (30 wt.% of the monomer mixture), and 6 wt. parts of the aliphatic urethane acrylate (per 100 wt. parts of a telomer syrup).

## Figures and Tables

**Table 1:** Composition of acrylic copolymers and fluorotelomers syrups.

Fluorotelomers or Acrylic Copolymers Components (wt. parts)					
BA	BMA	LMA	TFEtOH *	APO *	Syrup Acronym
65	15	20	0	0.5	AS/L20
55		30	0	0.5	AS/L30
45		40	0	0.6	AS/L40
65		20	2.5	0.45	FT2.5/L20
55		30	2.5	0.5	FT2.5/L30
45		40	2.5	0.6	FT2.5/L40
65		20	5.0	0.45	FT5/L20
55		30	5.0	0.6	FT5/L30
45		40	5.0	0.65	FT5/L40

* wt. part/100 wt. part of the monomers mixture.

**Table 2:** Dynamic viscosity and solids content of the fluorotelomers and acrylic syrups, as well as the molecular weights of the fluorotelomers and acrylic copolymers.

Syrup Acronym	η (Pa·s)	SC (wt.%)	Mw (g/mol)	Mn (g/mol)	Mw/Mn
AS/L20	>>60	no data	120,000	24,000	5.9
AS/L30	6.5	74	107,000	22,000	4.9
AS/L40	6.2	73	92,000	19,000	4.8
FT2.5/L20	6.6	64	29,000	1500	1.9
FT2.5/L30	11.4	68	26,000	1400	1.9
FT2.5/L40	7.0	75	23,000	1200	1.9
FT5/L20	8.2	76	24,000	1300	1.8
FT5/L30	5.8	76	22,500	1300	1.7
FT5/L40	6.5	74	21,000	1200	1.7

## Data Availability

Not applicable.
